# Clinical Variables and Peripheral Biomarkers Associated with Substance-Induced Psychotic Disorder: Differences Related to Alcohol, Cannabis, and Psychostimulant Abuse

**DOI:** 10.3390/jpm14030325

**Published:** 2024-03-21

**Authors:** Martina Di Paolo, Antonia Calabrese, Guido Nosari, Valentina Ciappolino, Luisa Cirella, Alice Caldiroli, Enrico Capuzzi, Massimo Clerici, Massimiliano Buoli

**Affiliations:** 1 Department of Neurosciences and Mental Health, Fondazione IRCCS Ca’ Granda Ospedale Maggiore Policlinico, 20122 Milan, Italy; guido.nosari@policlinico.mi.it (G.N.); valentina.ciappolino@policlinico.mi.it (V.C.); massimiliano.buoli@unimi.it (M.B.); 2 Department of Pathophysiology and Transplantation, University of Milan, 20122 Milan, Italy; a.calabrese26@campus.unib.it; 3 Healthcare Professionals Department, Foundation IRCCS Ca’ Granda Ospedale Maggiore Policlinico, 20122 Milan, Italy; luisa.cirella@policlinico.mi.it; 4 Psychiatric Department, Azienda Socio Sanitaria Territoriale Monza, 20900 Monza, Italy; a.caldiroli@asst-monza.it (A.C.); e.capuzzi1@campus.unimib.it (E.C.); massimo.clerici@unimib.it (M.C.); 5 Department of Medicine and Surgery, University of Milano Bicocca, Via Cadore 38, 20900 Monza, Italy

**Keywords:** alcohol, cannabis, clinical variables, peripheral biomarkers, psychostimulants, substance-induced psychotic disorder

## Abstract

**Background:** The present retrospective observational study aims to identify differences in clinical features and peripheral biomarkers among patients affected by substance-induced psychotic disorder (SIPD) according to the primary substance of abuse. **Methods:** A sample of 218 patients was divided into three groups according to the type of consumed substance: alcohol, cannabis, and psychostimulants. The three groups were compared using one-way analyses of variance (ANOVAs) for continuous variables and χ^2^ tests for qualitative variables. After excluding the alcohol-induced psychotic disorder group, the same analyses were repeated. The statistically significant variables from these subsequent analyses were included in a binary logistic regression model to confirm their reliability as predictors of cannabis- or psychostimulant-induced psychotic disorder. **Results:** Psychotic cannabis abusers were younger (*p* < 0.01), with illness onset at an earlier age (*p* < 0.01). Alcohol consumers presented a longer duration of illness (*p* < 0.01), more frequent previous hospitalizations (*p* = 0.04) and medical comorbidities (*p* < 0.01), and higher mean Modified Sad Persons Scale scores (*p* < 0.01). Finally, psychostimulant abusers had a higher frequency of lifetime history of poly-substance use disorders (*p* < 0.01). A binary logistic regression analysis revealed that higher mean Brief Psychiatric Rating Scale scores (*p* < 0.01) and higher sodium (*p* = 0.012) and hemoglobin (*p* = 0.040) plasma levels were predictors of cannabis misuse in SIPD patients. **Conclusions:** Different clinical factors and biochemical parameters con be associated with SIPD according to the main substance of abuse, thus requiring specific management by clinicians.

## 1. Introduction

Substance-induced psychotic disorder (SIPD) is defined by the Diagnostic and Statistical Manual of Mental Disorders, Fifth Edition (DSM-5), as a psychiatric disorder characterized by hallucinations and/or delusions that arise during or soon after substance intoxication or withdrawal [1]. This condition occurs with a notable frequency in the general population (6.5 out of 100,000 people per year in accordance with the latest studies) [2]. Several agents can contribute to the onset of SIPD, including alcohol, psychostimulants like cocaine, and cannabis, and the number of substances associated with this condition is continuously expanding [3,4].

The management of this condition can be challenging for clinicians for several reasons, including the fact that SIPD does not present stable diagnostic symptoms: approximately one-third of these patients are re-classified in the following years as subjects affected by schizophrenia or bipolar disorder [5]. Of note, one of the most important risk factors for the development of psychotic symptoms in people consuming recreational substances is having a family history of schizophrenia spectrum disorders [6,7].

A differential diagnosis between a primary psychotic disorder with comorbid substance misuse or SIPD and other psychotic disorders can therefore be difficult, potentially leading to delays in providing appropriate treatment for patients [8,9].

Some literature data suggest that different substances of abuse may confer a variable risk of SIPD [10] and may be associated with specific clinical features [11]. Of note, cannabis users with SIPD appear to exhibit cognitive symptoms similar to those observed in subjects affected by schizophrenia [12], whereas methamphetamine users would exhibit predominantly positive symptoms [13]. Other authors observed that patients with cannabis-induced psychotic disorders were more likely to show schizophrenia-like symptoms compared to alcohol abusers [14], such as paranoia, hallucination, and negative symptoms. This makes the clinical differential diagnosis between the two conditions extremely complex, while patients with alcohol-induced psychotic disorders typically exhibit more intense depressive and anxiety symptoms and fewer negative and disorganized symptoms [15]. Additionally, cannabis-induced psychosis, compared to psychoses linked to other substances of abuse, is associated with the highest conversion rate to schizophrenia [5].

The different clinical presentation of SIPD in relation to a specific substance can be attributed to the distinct mechanisms of action of the abused substance and related biological alterations. In. the case of alcohol, psychotic symptoms are hypothesized to result from excessive activity of the dopamine system as well as from the rebound effects of the prolonged inhibition of N-methyl-D-aspartate (NMDA) glutamate receptors [16]. Furthermore, alcohol-induced psychotic disorders can be triggered by neuronal membrane damage, thiamine deficiency, and gene expression modifications driven by increased histone acetylation [17]. Tetrahydrocannabinol (THC), the component of cannabis thought to be responsible for the onset of psychotic symptoms, has various biological effects, including the over-activation of the dopamine system, the inhibition of brain-derived neurotrophic factor (BDNF), and the modulation of NMDA and amino-3-hydroxy-5-methyl-4-isoxyzolepropionic acid (AMPA)-type glutamate receptors [18,19]. Similarly, the use of psychostimulants like cocaine increases the risk of psychotic disorders as a result of an imbalance in the dopamine system [20]. Additionally, recent research highlighted the role of inflammatory and oxidative processes as well as the activation of metabolic pathways resulting in alterations in neurotransmitter release [21,22]. It is worth noting that SIPD has been associated with several immunological alterations and that the various recreational drugs have different effects on biological systems. Similarly to what happens with schizophrenia, exocannabinoids favor the shift of T helper (Th) lymphocytes from subtype 1 to 2, enhancing humoral immunity and reducing acquired immunity activity [23,24]. Conversely, alcohol intake increases the levels of pro-inflammatory cytokines associated with both cell-mediated immunity and the innate response through the activation of innate immune receptor toll-like receptor 4 (TLR4) signaling in glial cells, which are supposed to increase the susceptibility to mood and anxiety symptoms rather than to psychotic ones [25,26]. Cocaine and methamphetamine have been associated with increased levels of pro-inflammatory cytokines [21,27] and to the activation of microglia. Cocaine abusers also exhibit epigenetic modifications of genes involved in neuroplasticity and innate immunity [28,29]. Preliminary data also suggest that recreational drugs can modify the microbiome, thereby facilitating the onset of several psychiatric conditions, including psychotic disorders [30]. Despite these findings, the current literature does not provide a clear identification of peripheral biological parameters that can indicate the prognosis of SIPD patients based on the type of abused substance.

In this framework, the present study has the objective to identify differences in clinical features and biochemical parameters between patients affected by SIPD classified according to the primary substance of abuse. The findings of the present research can assist clinicians in personalizing treatment strategies for patients affected by SIPD, also considering the limited data available in the literature on this topic.

## 2. Materials and Methods

In this retrospective observational study, we enrolled a sample of 218 patients consecutively admitted with a diagnosis of SIPD according to the DSM at the psychiatric inpatient clinics of Fondazione IRCCS Policlinico (Milan) and San Gerardo Hospital (Monza) from 2000 to 2022. The patients selected for the study had the following diagnosis at hospital discharge, according to the International Classification of Diseases, tenth edition, ICD-10: “mental and behavioral disorders due to the use of alcohol”, “mental and behavioral disorders due to the use of cannabinoids”, “mental and behavioral disorders due to the use of cocaine”, “mental and behavioral disorders due to the use of other stimulants”, and “unspecified non-organic psychosis” in comorbidity with a diagnosis of “psychoactive substance abuse”. The diagnosis of SIPD was made by an expert psychiatrist and, in the case of multiple hospitalizations, only the last admission was taken into consideration. The inclusion criteria were age ≥ 18 years and the abovementioned diagnosis. The exclusion criteria were the following: (1) ongoing treatment with pharmacological compounds that can exacerbate psychotic symptoms (e.g., corticosteroids or levetiracetam); (2) presence of medical comorbidities that can trigger the onset of psychotic symptoms (e.g., encephalitis or dementia) or significantly modify biochemical parameters (e.g., severe autoimmune diseases); (3) being in the perinatal period (pregnancy and one month postpartum), as this period is characterized by specific clinical features and biological changes in new mothers [31]. Patients with psychiatric comorbidities were included if SIPD represented the main psychiatric condition defined as the disorder primarily causing social dysfunction. On the first day of admission, socio-demographic and clinical data were obtained from clinical charts or interviews with patients and their relatives if information was not available. Each patient underwent a comprehensive psychometric assessment, evaluating global functioning and symptom severity, assessed, respectively, through the Global Assessment of Functioning (GAF) scale and the Positive and Negative Syndrome Scale (PANSS), together with the Brief Psychiatric Rating Scale (BPRS). Suicide risk was evaluated by the Modified Sad Persons Scale (MSPS), and aggressive behavior through the Modified Overt Aggression Scale (MOAS). Biochemical parameters were retrieved by intranet hospital applications, by examining blood analyses conducted at the beginning of hospitalization.

Of note, in our analyses the duration of illness refers to the duration of substance use disorder, and the duration of untreated illness (DUI) is defined as the time between the onset of psychotic symptoms and the administration of an antipsychotic compound [32].

The protocol of this study was reviewed and approved by the local ethic committee (approval number 1789).

Statistical analyses were performed through the Statistical Package for the Social Sciences (SPSS) for Windows (version 27.0). Descriptive analyses were performed on the total sample.

Three groups were identified according to the main substance of abuse (alcohol, cannabis, psychostimulants).

The psychostimulant-induced psychosis group included patients with an ICD10 diagnosis of “mental and behavioral disorders due to the use of cocaine” and “mental and behavioral disorders due to the use of other stimulants”. These two diagnoses were combined due to the small sample of patients presenting with other stimulant-induced psychoses and their similar clinical presentation, characterized by behavioral abnormalities, hallucinations, and paranoid delusions [33].

The three groups were compared by one-way analyses of variance (ANOVAs) for continuous variables and χ^2^ tests for qualitative variables. ANOVAs were then performed to compare cannabis and psychostimulant abusers for continuous variables. The statistically significant factors identified in this latter analysis were included in a binary logistic regression model as independent variables (predictors); the dependent variable was represented by cannabis versus psychostimulant users with psychotic disorders. This approach was chosen to exclude variables that were not already significantly different between cannabis and psychostimulant users in the univariate analyses, considering the large number of variables compared between the two groups. The quality of the model was evaluated by the Omnibus and Hosmer–Lemeshow tests. Statistical significance was set at *p* ≤ 0.05.

## 3. Results

The total sample included 218 patients, with the main substance of abuse being alcohol (N = 31), psychostimulants (most cocaine) (N = 71), and cannabis (N = 116). The clinical characteristics and biochemical parameters of the total sample and of the three groups identified by the main substance of abuse are displayed, respectively, in Table 1 and Table 2. Data on pharmacotherapy during hospitalization were available for 156 patients, and the main pharmacological compound prescribed during hospitalization were risperidone (N = 5), haloperidol (N = 56), paliperidone (N = 16), olanzapine (N = 27), quetiapine (N = 11), aripiprazole (N = 14), zuclopenthixol (N = 19), clozapine (N = 2), levomepromazine (N = 3), chlorpromazine (N = 1), promazine (N = 1), and clotiapine (N = 1). There were no significant differences in the frequency of prescription of a specific type of antipsychotic medication among the three groups (χ^2^ = 17.90, *p* = 0.71).

Univariate analyses revealed that psychotic cannabis abusers (compared to the other two groups) were younger (F = 25.29, *p* < 0.01), presented illness onset at an earlier age (F = 9.08, *p* < 0.01), and were more frequently tobacco smokers (χ^2^ = 11.26, *p* < 0.01). On the contrary, psychotic alcohol consumers were found to have a longer duration of illness (F= 8.43, *p* < 0.01), a higher number of previous hospitalizations (F = 3.36, *p* = 0.04), a higher prevalence of medical comorbidities (χ^2^ = 11.82, *p* < 0.01) and comorbidity with hypercholesterolemia (χ^2^ = 8.94, *p* = 0.01), higher mean MSPS scores (F = 6.19, *p* < 0.01), as well as higher plasma levels of total cholesterol (F = 3.98, *p* = 0.02), urea (F = 3.79, *p* = 0.03), and triglycerides (F = 3.69, *p* = 0.03). Lastly, psychotic psychostimulant abusers exhibited a higher frequency of lifetime history of poly-substance use disorders compared to the other two groups (χ^2^ = 27.34, *p* < 0.01). No other statistically significant differences were found between the three groups (*p* > 0.05).

The comparison between cannabis and psychostimulant abusers showed that the first group (compared to the second one) was younger (F = 12.13, *p* < 0.01), reported illness onset at an earlier age (F = 10.87, *p* < 0.01), and had lower GGT plasma levels (F = 4.08, *p* = 0.04) and higher red blood cell count (F = 4.94, *p* = 0.03) as well as sodium (F = 3.76, *p* = 0.05), hemoglobin (F = 5.49, *p* = 0.02), and albumin plasma levels (F = 6.22, *p* = 0.01). Furthermore, psychotic cannabis abusers demonstrated a trend to have higher mean BPRS scores (F = 3.21, *p* = 0.07) and a higher number of previous hospitalizations (F = 3.59, *p* = 0.060) compared to the other group. No other statistically significant differences were found between the two groups (*p* > 0.05).

The logistic regression model resulted to be reliable (Hosmer–Lemeshow test: χ^2^ = 9.011, *p* = 0.341; Omnibus test: χ^2^ = 31.039, *p* < 0.001), allowing for a correct classification of 76.4% of the cases. Psychotic cannabis abusers resulted to have higher mean BPRS scores (*p* < 0.01) as well as higher Na (*p* = 0.012) and hemoglobin (*p* = 0.040) plasma levels than psychotic stimulant abusers (Table 3).

## 4. Discussion

This study provides valuable insights into the clinical and biochemical features of individuals with substance-induced psychotic disorder (SIPD) based on their primary substance of abuse. The findings have several clinical implications, which will be discussed in detail in the following paragraphs.

Among the three groups categorized by the main substance of abuse, alcohol consumers resulted to be more prone to dyslipidemia and at higher suicide risk, as showed by their higher MSPS scores. The development of dyslipidemia in alcohol abusers can be attributed to the specific metabolism of alcohol, which leads to the accumulation of lactic acid and a block of the Krebs’ cycle. This, in turn, is associated with the transformation of acetyl-CoA excess in fatty acids [34]. The detrimental physical effects of alcohol consumption [35] also contribute to the higher frequency of medical comorbidities observed in alcohol abusers compared to the other two groups. Notably, the mean plasma triglyceride levels in our sample of individuals with alcohol misuse exceeded the recommended threshold of 150 mg/dL [36]. In a previous study, drug-naive patients with psychotic disorders showed higher total cholesterol and triglyceride plasma levels than healthy controls [37]. The similar elevation in urea levels in alcohol chronic abusers can be explained by a metabolic counterresponse to the downregulation of the urea cycle during alcohol intoxication [14]. Furthermore, the higher risk of self-harm in the alcohol group aligns with evidence indicating a 94% increase in death by suicide among individuals with alcohol use disorders [38]. Additionally, alcohol abusers frequently suffer from concomitant anxiety and depressive disorders [39] and often face conditions of social exclusion [40].

Regarding the patients with SIPD and cannabis misuse, this group showed a more severe clinical presentation (higher BPRS scores) than the patients with cocaine abuse, in agreement with previous literature [41]. Cannabis-induced psychotic disorders are often the result of the early consumption of forms of substances with high THC content [42], and this aspect can explain the young age and early age at illness onset of the patients with SIPD and cannabis abuse. Moreover, the earlier age of onset of psychotic symptoms in cannabis abusers underscores the importance of differentiating between transient intoxication-induced psychotic disorders and a stable psychotic disorder. Factors associated with the development of SIPD in cannabis users include continuous and early cannabis consumption, and cannabis was found to be more closely associated with the conversion of a psychotic disorder to schizophrenia compared to other recreational drugs [5]. The finding of an earlier age of onset of psychotic symptoms in cannabis abusers might suggest a higher risk of developing psychotic symptoms in this specific population, particularly if cannabis consumption begins during adolescence. Moreover, psychotic cannabis abusers were much more frequently found to be tobacco smokers than the patients in the other two groups. This may be explained by the fact that smoking is one of the most popular ways to consume cannabis, but cannabis is also able to potentiate the reward effects of nicotine [43]. Of note, the lack of CB1 cannabinoid receptors in mice led to reduced reward after acute nicotine administration [44]. Regarding the biochemical parameters, psychotic cannabis abusers showed higher hemoglobin levels compared to psychostimulant abusers. In agreement with this finding, a recent article reported that cannabis can increase the hemoglobin plasma levels as a result of hemolysis [45]. This hypothesis is also supported by the fact that the mean blood levels of hemoglobin appeared to be lower in drug-naive patients affected by psychotic disorders than in healthy controls [46]. Similarly, we found higher albumin plasma levels in cannabis abusers compared to psychostimulant abusers. Albumin has antioxidant properties [47], and previous research reported that higher levels of this molecule are associated with a better prognosis of psychiatric disorders [48,49]. In addition, lower albumin plasma levels were detected in ultra-high-risk psychotic patients who converted to a condition of full-blown psychotic disorders with respect to those who did not; thus, this parameter could be monitored to predict the risk of psychotic disorders in vulnerable subjects [50].

Finally, psychotic psychostimulant abusers more frequently showed poly-substance use disorders than the other two groups. This finding agrees with the available literature reporting that cocaine or other psychostimulant abusers frequently counterbalance the effects of these substances by recurring to central nervous system depressants such as alcohol or opioids [51]. The sodium levels were in the physiological range for both psychotic cannabis and psychostimulant abusers, but the significant differences between the two groups could be interpreted by the fact that psychostimulants cause excessive water intake and inappropriately elevated antidiuretic hormone (ADH) levels [52]. Of note, the sodium levels may be seen as an indirect indicator of adrenocorticotropic hormone (ACTH) plasma levels, which were reported to be higher in drug-naive first-episode psychotic patients than in healthy controls [53]. Moreover, the elevation in GGT plasma levels in psychostimulant abusers is the result of liver metabolism by cytochrome P450 and the production of pro-oxidant compounds [54].

The findings of the present article indicate that patients with SIPD may require a different management depending on the type of the main abused substance. In patients with alcohol-induced psychotic disorder, close monitoring of the lipid profile would be useful for the early identification of medical complications and to propose supportive interventions to reduce the suicide risk. In subjects with cannabis-induced psychotic disorder, the management of psychiatric symptoms may be more challenging, and blood counts should be monitored. In the case of comorbidity with smoking, interventions aimed at reducing cigarette smoking could be useful (e.g., nicotine patch or varenicline) [55]. In patients with psychostimulant-induced psychotic disorder, the concomitant use of other substances should be investigated, and access to therapeutic programs focused on these aspects should be encouraged.

It is interesting to note that some of the biochemical parameters measured in this study were designed to predict the prognosis of patients at ultra-high psychotic risk [56]. As mentioned above, lower albumin plasma levels were detected in high-risk psychotic subjects who converted to a condition of full-blown psychotic disorder compared to those who did not [50]. In addition, low cholesterol plasma levels predicted future suicide attempts in subjects with recent-onset psychotic disorders [57]. Some of these biochemical parameters could be implemented to predict and therefore prevent the onset of psychotic disorders in subjects at risk, and for these reasons further research in this area is desirable.

These results must be read in light of different limitations identified in our study, including the following: (1) the retrospective design; (2) the use of routinely investigated biochemical parameters during hospital admissions, without a preliminary selection; (3) missing data for certain variables, either because the information was not routinely collected in one of the two inpatient clinics, or because it was impossible to deduce it from the medical records; (4) the inclusion of patients who presented at hospital in a state of intoxication or active abuse, but not of patients who presented in a state of alcohol or substance withdrawal; (5) the lack of differentiation between different psychostimulant substances.

The findings of the present study suggest that patients with SIPD can require a specific management according to the primary substance of abuse. Future studies with larger samples are necessary to confirm the present findings and identify the optimal management of these patients. 

## Figures and Tables

**Table 1:** Demographic and clinical variables of the total sample and of the three groups defined according to the substance of abuse.

Variables		Total Sample N = 218	Alcohol-Induced Psychosis N = 31 (14.2%)	Psychostimulant-Induced Psychosis N = 71 (32.6%)	Cannabis-Induced Psychosis N = 116 (53.2%)	F or χ^2^	*p*-Value
Gender Missing = 0	*Male*	191 (87.6%)	28 (90.3%)	59 (83.1%)	104 (89.7%)	1.99	0.40
	*Female*	27 (12.4%)	3 (9.7%)	12 (16.9%)	12 (10.3%)		
Age (years) Missing = 0		33.89 (±12.21)	45.42 (±13.64)	35.41 (±11.91)	29.87 (± 9.63)	25.29	**<0.01**
Age at illness onset (years) Missing = 15		28.09 (±10.97)	33.28 (±12.90)	30.38 (±11.58)	25.20 (±9.07)	9.08	**<0.01**
Duration of hospitalization (days) Missing = 60		11.59 (±9.57)	8.92 (±6.38)	13.63 (±12.51)	10.99 (±7.57)	2.43	0.09
Duration of untreated illness (years) Missing = 61		1.18 (±2.74)	0.63 (±1.61)	1.36 (±3.19)	1.23 (±2.68)	0.62	0.54
Duration of SUD (years) Missing = 15		5.83 (±8.81)	11.83 (±14.64)	4.97 (±7.42)	4.74(±6.75)	8.43	**<0.01**
Presence of previous hospitalizations Missing = 8		133 (63.3%)	21 (67.8%)	39 (57.4%)	73 (65.8%)	1.59	0.45
Number of previous hospitalizations Missing = 8		2.18 (±4.73)	3.71 (±7.05)	1.71 (±1.48)	2.38 (±5.13)	3.36	**0.04**
Presence of family history of psychiatric disorders Missing = 60		55 (34.8%)	10 (40.0%)	13 (23.2%)	32 (41.6%)	5.16	0.08
Presence of family history of multiple psychiatric disorders Missing = 60		33 (20.9%)	5 (20.0%)	10 (17.9%)	18 (23.4%)	0.61	0.74
Presence of family history of substance use disorders Missing = 60		24 (15.2%)	4 (16.0%)	12 (21.4%)	8 (10.4%)	3.08	0.20
Presence of lifetime history of poly-substance use disorders Missing = 0		118 (54.1%)	7 (22.6%)	54 (76.1%)	57 (49.1%)	27.34	**<0.01**
Presence of tobacco smoke Missing = 5		106 (48.6%)	15 (48.4%)	24 (34.3%)	67 (59.8%)	11.26	**<0.01**
Current prescription of benzodiazepines Missing = 60		120 (75.8%)	15 (60.0%)	46 (82.1%)	59 (76.7%)	4.68	0.10
Current treatment with more than one psychotropic drug Missing = 60		153 (96.8%)	23 (92.0%)	54 (96.4%)	76 (98.7%)	2.81	0.25
Comorbidity with at least one psychiatric diagnosis Missing = 60		66 (41.8%)	12 (48.0%)	25 (44.6%)	29 (60.4%)	1.12	0.60
Comorbidity with more than one psychiatric diagnosis Missing = 60		21 (13.2%)	5 (20.0%)	6 (10.7%)	10 (12.9%)	1.31	0.50
Presence of comorbid personality disorders Missing = 60		37 (17.0%)	3 (12%)	16 (28.6%)	18 (23.4%)	2.65	0.27
Presence of lifetime suicide attempts Missing = 0		29 (13.3%)	6 (19.4%)	10 (14.1%)	13 (11.2%)	1.46	0.48
Number of lifetime suicide attempts Missing = 0		0.17 (±0.56)	0.23 (±0.50)	0.18 (±0.66)	0.16 (±0.50)	0.21	0.81
Comorbidity with other medical conditions Missing = 60		69 (59.2%)	17 (68.0%)	28 (50.0%)	24 (31.2%)	11.82	**<0.01**
Comorbidity with multiple medical conditions Missing = 60		23 (14.6%)	6 (24.0%)	8 (14.3%)	9 (11.7%)	2.31	0.32
Presence of hypothyroidism Missing = 60		9 (5.7%)	0 (0.0%)	6 (10.7%)	3 (3.9%)	4.60	0.10
Presence of hypercholesterolemia Missing = 0		32 (14.7%)	10 (32.3%)	8 (11.3%)	14 (12.1%)	8.94	**0.01**
Presence of diabetes Missing = 60		11 (7.0%)	3 (12.0%)	6 (10.7%)	2 (2.6%)	4.46	0.11
Presence of obesity Missing = 0		5 (2.3%)	1 (3.2%)	1 (1.4%)	3 (2.6%)	0.41	0.81
Lifetime psychotherapy Missing = 60		12 (7.6%)	1 (4.0%)	2 (3.6%)	9 (11.7%)	3.59	0.17
History of obstetric complications Missing = 0		25 (11.5%)	5 (16.1%)	4 (5.6%)	16 (13.8%)	3.66	0.16
GAF score Missing = 61		46.37 (±15.20)	58.04 (±15.83)	53.71 (±16.66)	54.87 (±13.3)	0.73	0.49
PANSS score Missing = 61		61.83 (±15.16)	60.76 (±15.33)	59.45 (±16.49)	63.93 (±13.94)	1.50	0.23
BPRS score Missing = 46		43.97 (±12.13)	46.61 (±11.62)	41.27 (±12.04)	44.96 (±12.16)	2.54	0.08
MSPS score Missing = 61		2.49 (±1.09)	3.16(±1.21)	2.29 (±1.02)	2.42 (±1.04)	6.19	**<0.01**
MOAS score Missing = 1		4.34 (±4.91)	4.03 (±5.04)	4.72 (±4.88)	4.19 (±4.93)	0.32	0.73

Legend: BPRS = Brief Psychiatric Rating Scale; GAF = Global Assessment of Functioning; MOAS = Modified Overt Aggression Scale; MSPS = Modified Sad Persons Scale; PANSS = Positive and Negative Syndrome Scale; SUD = Substance Use Disorder. Means for quantitative variables and frequencies for qualitative ones are reported. Standard deviations for quantitative variables and percentages for qualitative variables are reported into brackets. In bold, statistically significant *p* value resulting from chi-square tests (χ^2^) or analyses of variance (F) (*p* ≤ 0.05).

**Table 2:** Biological variables of the total sample and of the three groups defined according to the substance of abuse.

Variables	Total Sample N = 218	Alcohol-Induced Psychosis N = 31 (14.2%)	Psychostimulant-Induced Psychosis N = 71 (32.6%)	Cannabis-Induced Psychosis N = 116 (53.2%)	F	*p*-Value
Sodium (Na) (mEq/L) Missing = 84	141.45 (±2.50)	142.08 (±2.71)	140.80 (±2.90)	141.70 (±2.00)	2.66	0.07
Potassium (K) (mEq/L) Missing = 84	4.23 (±0.38)	4.26 (±0.41)	4.24 (±0.34)	4.21 (±0.39)	0.22	0.81
Na/K ratio Missing = 86	33.72 (±3.04)	33.69 (±3.17)	33.47 (±2.64)	33.91 (±3.28)	0.29	0.75
Number of lymphocytes (10^9^/L) Missing = 64	2.58 (±1.59)	2.08 (±0.68)	2.55 (±0.76)	2.75 (±2.07)	1.70	0.19
Number of neutrophils (10^9^/L) Missing = 64	5.00 (±2.60)	4.98 (±2.51)	5.13 (±2.74)	4.93 (±2.57)	0.09	0.91
NLR Missing = 77	2.19 (±1.32)	2.77 (±2.03)	2.01(±1.09)	2.13(±1.17)	2.47	0.09
Number of RBCs (10^12^/L) Missing = 25	4.86 (±0.57)	4.81 (±0.61)	4.74 (±0.57)	4.94 (±0.54)	2.52	0.08
Number of WBCs (10^9^/L) Missing = 25	8.56 (±3.11)	8.15 (±2.56)	8.68 (±3.19)	8.60 (±3.22)	0.30	0.75
MCV (fL) Missing = 86	87.61 (±6.85)	89.65 (±9.36)	87.43 (±6.27)	87.03 (±6.21)	1.22	0.30
HB (g/dL) Missing = 29	14.49 (±1.55)	14.36 (±1.68)	14.15 (±1.62)	14.73 (±1.44)	2.77	0.07
Number of PLTs (10^9^/L) Missing = 79	254.13 (±85.54)	259.73 (±122.87)	265.23 (±71.31)	244.91 (±80.44)	0.85	0.43
MPV (fL) Missing = 86	10.57 (±1.10)	10.28 (±0.90)	10.67 (±1.21)	10.60 (±1.08)	0.97	0.38
Glycemia (mg/dL) Missing = 27	90.37 (±22.79)	93.75 (±25.21)	91.21 (±25.10)	88.91 (±20.62)	0.55	0.58
Creatinine (mg/dL) Missing = 32	0.90 (±0.15)	0.89 (±0.16)	0.92 (±0.16)	0.89 (±0.15)	0.68	0.51
Urea (mg/dL) Missing = 63	27.64 (±9.08)	31.56 (±11.03)	27.92 (±10.23)	26.10 (±7.03)	3.79	**0.03**
Uric acid (mg/dL) Missing = 96	5.50 (±1.60)	6.06 (±1.29)	5.18 (±1.46)	5.50 (±1.76)	2.16	0.12
ALT (U/L) Missing = 29	32.19 (±30.43)	31.59 (±28.20)	36.90 (±41.76)	29.60 (±22.05)	1.08	0.34
AST (U/L) Missing = 59	39.68 (±45.91)	40.81 (±54.97)	39.34 (±40.69)	39.51 (±46.07)	0.01	0.99
GGT (U/L) Missing = 36	29.69 (±38.97)	33.54 (±32.40)	37.38 (±60.32)	24.12 (±18.83)	2.29	0.10
Bilirubin (mg/dL) Missing = 44	0.68 (±0.42)	0.61 (±0.30)	0.61 (±0.39)	0.73 (±0.46)	1.77	0.17
Total plasmatic proteins (g/dL) Missing = 97	6.88 (±0.55)	6.91 (±0.54)	6.84 (±0.66)	6.89 (±0.49)	0.16	0.85
Albumin (g/dL) Missing = 87	4.42 (±0.46)	4.38 (±0.59)	4.30 (±0.44)	4.51 (±0.40)	2.74	0.07
Total cholesterol (mg/dL) Missing = 57	170.72 (±44.23)	192.92 (±49.51)	169.00 (±53.78)	165.31 (±34.16)	3.98	**0.02**
Triglycerides (mg/dL) Missing = 128	112.96 (±77.20)	159.13 (±112.31)	102.55 (±65.15)	103.13 (±64.29)	3.69	**0.03**
LDH (mU/mL) Missing = 96	207.61 (±94.58)	205.02 (±102.96)	220.38 (±110.85)	199.34 (±77.81)	0.61	0.55
CPK (U/L) Missing = 54	511.76 (±890.42)	292.82 (±366.90)	392.79 (±530.87)	674.59 (±1163.05)	2.11	0.13
PChE (U/L)Missing = 104	7523.62 (±2084.10)	8001.14 (±2115.56)	7431.03 (±1654.13)	7405.73 (±2323.45)	0.67	0.51
TSH (mcU/mL) Missing = 111	1.81 (±1.35)	1.51 (±0.85)	2.03 (±1.73)	1.78 (±1.22)	0.99	0.37

Legend: ALT = alanine transaminase; AST = aspartate transaminase; CPK = creatine phosphokinase; GGT = gamma-glutamyl transferase; HB = hemoglobin; LDH = lactate dehydrogenase; MCV = mean corpuscular volume; MPV = mean platelet volume; NLR = neutrophil-to-lymphocyte ratio; PChE = pseudocholinesterase; PLTs = platelets; RBCs = red blood cells; TSH = thyroid-stimulating hormone; WBCs = white blood cells. Means and standard deviations (into brackets) are reported. In bold, statistically significant *p* value resulting from ANOVAs (*p* ≤ 0.05).

**Table 3:** Binary logistic regression model with predictors of cannabis versus psychostimulant users with psychotic disorders.

Variables	B	S.E.	Wald	*p*	OR	95% CI for OR
Age at hospital admission	−0.064	0.042	2.343	0.126	0.938	0.864–1.018
Age at illness onset	0.018	0.043	0.171	0.679	1.018	0.936–1.106
Number of previous hospitalizations	0.221	0.172	1.651	0.199	1.248	0.890–1.748
BPRS score	0.082	0.028	8.8606	**0.003**	1.085	1.027–1.146
Sodium (Na)	0.343	0.137	6.308	**0.012**	1.409	1.078–1.842
Number of RBCs	−0.324	0.567	0.327	0.567	0.723	0.238–2.198
Hb	0.440	0.214	4.222	**0.040**	1.553	1.021–2.362
GGT	−0.014	0.014	1.048	0.306	0.986	0.959–1.013
Albumin	0.735	0.686	1.147	0.284	2.085	0.543–7.999

Legend: B = regression coefficient; BPRS = Brief Psychiatric Rating Scale; CI = confidence interval; GGT = gamma-glutamyl transferase; Hb = hemoglobin; OR = odds ratio; RBCs = red blood cells; S.E. = standard error of B; Wald = Wald statistics. Dependent variable is represented by cannabis versus psychostimulant users. In bold, statistically significant *p* value (≤0.05).

## Data Availability

Data are available under request to the corresponding author.
